# WebTag: Web Browsing into Sensor Tags over NFC

**DOI:** 10.3390/s120708675

**Published:** 2012-06-26

**Authors:** Juan Jose Echevarria, Jonathan Ruiz-de-Garibay, Jon Legarda, Maite Álvarez, Ana Ayerbe, Juan Ignacio Vazquez

**Affiliations:** 1 Deusto Institute of Technology–DeustoTech, University of Deusto, Avenida de las Universidades 24, Bilbao 48007, Spain; E-Mail: jlegarda@deusto.es; 2 Information & Interaction Systems, Tecnalia, Parque Tecnológico de Bizkaia, Zamudio 48170, Spain; E-Mails: maite.alvarez@tecnalia.com (M.A.); ana.ayerbe@tecnalia.com (A.A.); 3 Department of Telecommunications, University of Deusto, Avenida de las Universidades 24, Bilbao 48007, Spain; E-Mail: ivazquez@deusto.es

**Keywords:** wireless sensor monitoring, near field communication, wireless sensor networks, internet of things, embedded web server, TCP/IP tunneling, sensor tag

## Abstract

Information and Communication Technologies (ICTs) continue to overcome many of the challenges related to wireless sensor monitoring, such as for example the design of smarter embedded processors, the improvement of the network architectures, the development of efficient communication protocols or the maximization of the life cycle autonomy. This work tries to improve the communication link of the data transmission in wireless sensor monitoring. The upstream communication link is usually based on standard IP technologies, but the downstream side is always masked with the proprietary protocols used for the wireless link (like ZigBee, Bluetooth, RFID, *etc.*). This work presents a novel solution (WebTag) for a direct IP based access to a sensor tag over the Near Field Communication (NFC) technology for secure applications. WebTag allows a direct web access to the sensor tag by means of a standard web browser, it reads the sensor data, configures the sampling rate and implements IP based security policies. It is, definitely, a new step towards the evolution of the Internet of Things paradigm.

## 1. Introduction

Sensor monitoring had seen its own technological revolution in the low scaling of the wireless communication technologies. As soon as the electronic technology was able to offer good performance—in terms of power consumption and cost—in analog to digital converters (ADC), data processing units and radiofrequency front ends, wireless embedded systems were integrated with sensor technologies for a non-intrusive monitoring [1]. The result is nowadays known as *wireless sensor networks* (WSNs) and it is considered one of the key topics in communication engineering [2].

A WSN is made up of distributed devices (known as nodes or tags) with some kind of sensing capability to monitor physical or environmental parameters. They are connected wirelessly creating different type of networks based on the IP wired technology and they cover a large range of application areas, mainly when power or infrastructure limitations make a wired solution difficult to use.

Despite the maturity of the WSNs, there are still many challenges to overcome, like the increase of the computational power of the sensor devices with lower power requirements [3]; more robust encryption [4] and security techniques [5], an energy efficiency increase [6], new network architectures [7], better network deployment [8] or more efficient data routing strategies [9] and communication protocols [10].

The interaction within WSNs, *i.e.*, how the sensor information is transmitted and processed until the decision is made, has many variations depending on each application. Traditionally, sensor data is read and transmitted immediately through the wireless network to a dedicated gateway, which processes the information or retransmits it upstream to a remote smart device. This is known as *remote* monitoring. However, there is an alternative whereby the data processing is done by a dedicated reader located next to the sensor tag, and this can be called a *near field* monitoring.

In both cases, there is a data transmission phase that is done with standard TCP/IP protocols, but there is another one that always depends on the technology used in the wireless link. This work tries to take one step ahead in the interaction within WSNs, proposing an integral web interaction from the end user to the sensor tags in order to take advantage of the IP network technologies during all the data transmission process. In other words, we put each sensor tag directly in the Internet as part of the Internet of Things paradigm [11], whereby everything will be interconnected anytime and anywhere.

The primary purpose of the current paper is to present the new concept of near field sensor tags with a web browsing interaction for secure applications. This is done by tunneling the TCP/IP traffic over a near field radiofrequency technology (NFC), avoiding the use of proprietary protocols and application program interfaces. As the NFC identification technology is not intended to exchange large amount of data, the problems found so far and the way they have been solved, as well as the advantages of this near field web access approach, will be shown in the next sections.

The remainder of the paper is organized as follows: Section 2 reviews the basic access modes of sensor monitoring used nowadays and the main wireless technologies used. Section 3 introduces the concept of the proposed sensor tag with a web access. Section 4 presents a summarized explanation of the implementations and the system evaluation of the WebTag. The conclusions of this paper are summarized in Section 5.

## 2. Access Modes of Sensor Wireless Monitoring

Sensor wireless monitoring can be analyzed and classified in many ways, but in this section we are going to focus on one specific aspect: the access mode.

Regarding the interaction in sensor wireless monitoring, we can simplify it into two access modes: near field or remote access, depending on where the information is processed. In the first case, a dedicated device (*reader*) reads directly the sensor data, processes the information and interacts with the end user depending on the application requirements. However, in the second case, the sensor data is retransmitted through the wireless network before it reaches the network gateway, where that data is sent to an upper decision making system. The WSNs use mainly the remote access mode, while the near field mode is more used in classical wireless monitoring.

**Figure 1.**
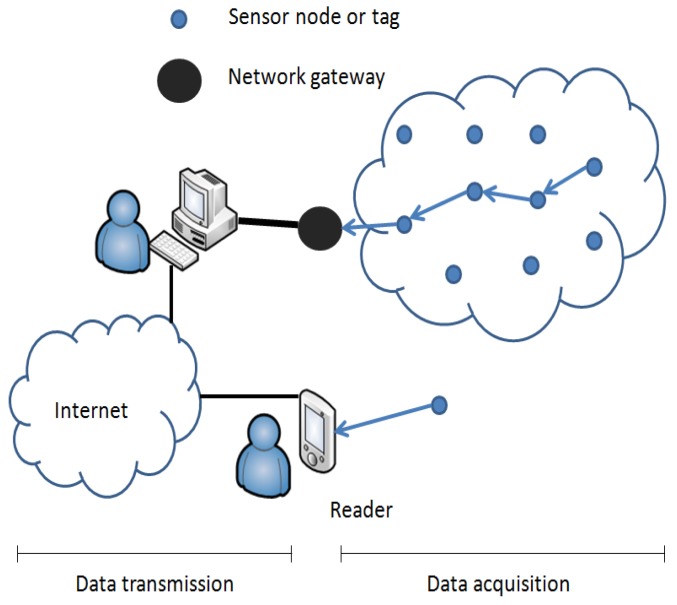
Remote and near field access modes.

In both cases, the communication process can be divided into two main stages: the former (on the right side of the Figure 1) is carried out when the data is transmitted from the sensor tag to the reader or the network gateway (let's define it as *data acquisition*), depending on the access mode. The latter, is done when data is sent upstream from the reader or the gateway node to an upper decision making system or to the Internet (let's call it *data transmission*). Usually data transmission is done with IP-based technologies, but the data acquisition stage depends on the wireless technology used, thus, the data is packaged according to the respective communication protocol used (ZigBee, Bluetooth, *etc.*). The reader or the network gateways are responsible for doing the conversion to the upstream IP network.

Let's focus on the data acquisition stage. Each communication technology specifies part of the communication protocols required for the data transmission [12], some more than others, but in all cases specific application program interfaces (APIs) are developed in order to manage the transmitted information in the reader or in the network gateway. This dependency on proprietary protocols reduces the flexibility of subsequent changes and the integration with other devices, limits the access to the sensor data to those devices with the same wireless interface and the specific API installed, and delegates the access security issues to the implemented encryption techniques. Using IP technologies in the data acquisition stage would allow a direct web based access to the sensor tag, solving all these disadvantages, as it provides a standard implementation for all the communication protocols.

Security issues have been briefly mentioned, but they have a key importance in this work. Regarding the access modes, both remote and near field modes have differences and common problems in security issues.

The near field access mode, as it is based on point-to-point communication links, allows authentication, encryption and compression methods in a more efficient way [13] than in remote access modes (WSNs) with multi-point links. However, the ubiquity of the wireless access is still a big challenge for both access modes, mainly for specific security applications [14]. The information can be captured by malicious users even if encryption techniques are used (not always available), so it is very difficult to guarantee the security of the communications [15]. For this type of applications, the very near field (point-to-point) access modes are chosen as the most secure interaction methods.

As the present work is focused on web based access for secure wireless sensor monitoring, let's go more in deep in topics like web access and near field point-to-point technologies.

### 2.1. Web Based Access

The web based access in the data acquisition stage of sensor wireless monitoring systems has increased due to the progressively use of Internet Protocol (IP) in computational and sensing devices [16], but mainly with non-proprietary technologies, like WiFi.

The IP for Smart Objects (IPSO) Alliance and the Internet Engineering Task Force (IETF) have recently promoted the use of IP in low power embedded systems in order to avoid proprietary standards. This way, in remote access systems, the data acquisition and transmission stages can be connected without any network gateway, and in near field solutions, the reader will not be dedicated anymore, as any web browser would be able to interact with the sensor tag using the standard TCP/IP communication protocols.

On the other hand, a standard suite of protocols like TCP/IP can improve the security performance by means of classical security solutions, like cryptography, web authentication, Secure Sockets Layer (SSL) and Transport Layer Security (TLS) for web traffic and IPsec for the network layer security. The fact of using the Internet based security techniques in WSNs is an added security improvement compared to the proprietary protocols.

Therefore, proprietary protocols are trying to enhance their application capabilities with native IP support. ZigBee for example is now supporting IP, seamlessly enabling the use of diverse devices (even from different vendors) without using a gateway translation layer.

Bluetooth is also moving towards IP tunneling [17] in order to extend the device connectivity. However, near field technologies that can be used for sensor wireless monitoring, like RFID or NFC, do not have IP support yet.

The challenge on this topic relies on the IP tunneling of proprietary near field wireless technologies over the tight performance requirements of the sensor tags: power consumption, computing capacities, bidirectional data transmission, costs, *etc.*

### 2.2. Near Field Point-to-Point Technologies

There are not many technologies that allow a near field point-to-point access for sensor monitoring, maybe radiofrequency identification (RFID) technology in all its variants (LF/HF/UHF/Microwave) is the most used since the passive radio link was merged with sensing and small computing tasks [18].

**Figure 2.**
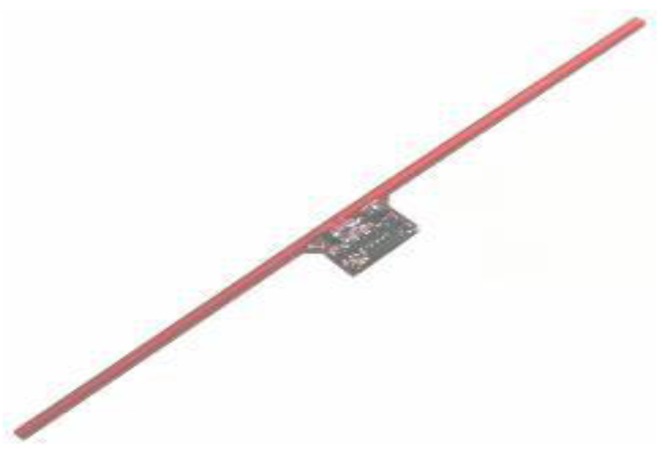
WISP.

One of best examples is Wireless Identification and Sensing Platform (WISP) [19], which integrates sensing and wireless communication in a passive device based on RFID technology (shown in Figure 2). WISP is entirely powered by the radio signal used in the communication link, harvesting the radiofrequency energy from the dedicated reader. All the sensor, control and communication modules are powered by the same source and it can perform many computing tasks, including sampling and data transmission.

The major advantages of these sensor tags are the ones directly inherited from the use of classic RFID: tiny, inexpensive and long-life tags, and the point-to-point and its simple communication procedure, where the tag is seen by the reader as a standard tag and the sensor data is attached to the identification string. There is also a growing tendency that tries to make these sensing RFID devices cooperate with wireless sensor networks [20] or even create their own networks [21], so that a more robust monitoring systems and new applications arise.

However, RFID sensing devices have many drawbacks. On the one hand, when no reader is present, the sensor tags have no power supply and therefore they cannot work, the data is not internally saved and obviously there is no radio communication link. In the other hand, they use proprietary communication protocols and *ad-hoc* user interfaces designed to access the stored data, which has no structured format. But the main disadvantage for the implementation of a web based interaction over RFID is the absence of bidirectional communication.

Bluetooth instead is a bidirectional, short-range and master-slave wireless technology working at 2.4 GHz and it is mainly used to connect computers, mobile phones, and handheld devices. Bluetooth is also a proprietary protocol, but it allows a bidirectional and point-to-point interaction, but needs of a pairing process to enable that communication link. Bluetooth has also been used to form wireless sensor networks using TCP/IP [22].

Near Field Communication (NFC) is another near field point-to-point wireless communication technology that is achieving importance in some sectors for user identification because of the bidirectional capacity. NFC works in the HF band (13.56 MHz) and it somehow extends and covers the capabilities of RFID, despite RFID is mainly applied to item, product or people tracking, and NFC has been used to provide contactless payment services [23] or other services with near field identification needs.

That is because RFID can operate to reading ranges of several meters, which could be not suitable for reliable applications. However, the NFC read range, of only a few centimeters, provides a good performance in high secure and reliable applications like mobile payments and access control, which need the exchange of creditable information.

The drawbacks of NFC are the greater complexity of the bidirectional communication and the need of batteries. Thus, systems with NFC readers provide broader use than RFID because NFC allows the deployment of new services based on its bidirectional (peer-to-peer) communication capacities [24], and a continuous sampling of the sensor despite the absence of the reader.

When compared with other point-to-point (see Table 1) and bidirectional technologies like Bluetooth, the main advantages of NFC are the instant connection between the two devices, without long pairing processes, and the very short read range which is a key aspect for secure applications. It also has less interferences and lower power consumption.

NFC is the best technology for high security applications, web based interaction and continuous monitoring, mainly because the near field point-to-point access, the bidirectional capability that allows the implementation of IP tunneling and the use of batteries. That is why NFC was the technology selected for the present work. The next section shows the WebTag concept for web access in NFC sensor tags.

## 3. WebTag

The main goal of WebTag is to modify the interaction and accessibility method of sensor tags to a web browsing model, reducing the impacts on the battery life, the computational requirements and the security threats regarding the ubiquity of the wireless access.

For this purpose, WebTag intends to merge the benefits of the accessing methods shown before. On the one hand, the security of the near field point-to-point access and on the other hand the usage of non proprietary protocols and user interfaces over the NFC technology. This approach will put the sensor tags directly in the Internet without using a protocol gateway, with all the advantages of web browsing and service invocation, improving the response time to an emergency, malfunction or damage of the sensor tag, and reducing the related costs. The basic requirements of WebTag can be classified as follows:

- Architecture
  - Embedded microcontroller
  - Optimized battery based design
  - Battery charger system onboard
  - Bidirectional radiofrequency communication (NFC)
  - Real Time Clock (RTC)
  - Set of sensors
- Communication Protocol
  - Use common and standardized communication protocols (TCP/IP)
- Processing Unit
  - Use of embedded web server to serve the data to the client side
- Security
  - Web access control methods aside encryption implementations
  - Header and data encryption
- Wireless Transmission
  - Minimize the information exchange due to the low rates and latencies of embedded systems
  - TCP/IP header compression
  - HTML data compression
- Power Consumption
  - Enable low power modes

In next sections some details are disclosed for each subject.

### 3.1. Architecture

**Figure 3.**
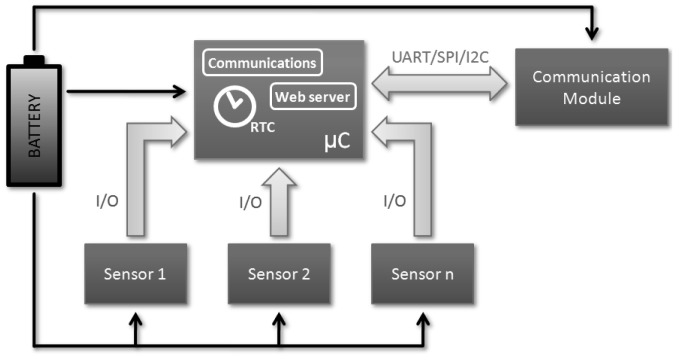
WebTag architecture.

The WebTag architecture (see Figure 3) does not differ much from the rest of existing sensor tags. The microcontroller is the *head* of the device, it controls the basic decisions like when do the measurement, where store the data, when do the transmission and what type of logic or arithmetic operation should be applied. The use of a Real Time Clock module (implemented by hardware or software) is very interesting in order to have a solid time reference, and the communications with the peripheral devices (memories, RF modules, sensors or acquisition devices) is done through different *interfaces* as Universal Asynchronous Receiver Transmitter (UART), Serial Peripheral Interface (SPI) or digital input/outputs.

Sensors are normally connected through conditioning circuits and these are completely application dependent. The physical principle, resolution, power and signal conditioning requirements define both microcontroller and battery specifications, and therefore the overall system life cycle, but from the architecture point of view, the use of standard interfaces and battery cells allows us to cover a wide range of sensors.

The communication technologies depends on the coverage, data rate, power and legal requirements, and the architecture depends on the communication procedure. For the NFC wireless solution the system needs to include a radiofrequency transceiver, an antenna and the matching circuitry between them.

Last but not least, it is mandatory the use of some kind of active power source, even its dimensioning is carefully optimized regarding the application duty cycle, the microcontroller operation modules, the communication strategy and the alternative power harvesting techniques. This is, along with the sensor technologies, one of the biggest challenges within the wireless sensor networks.

### 3.2. Communication Protocol

WebTag uses the Transmission Control Protocol/Internet Protocol (TCP/IP) communication protocol. It is the basic communication protocol used in the Internet and it is composed by two layers: TCP provides the communication with the application layer (HTTP) and IP layer handles the destination of each packet.

TCP/IP has a client/server model: a client asks a service petition trough the web browser, then a request is sent to the proper server, and the server (WebTag in this case) uses TCP/IP to respond to the browser. That means TCP/IP communication is primarily bidirectional and point to point, from the client side to the server side.

NFC is not intended to carry TCP/IP traffic. NFC uses a master-slave model and small payloads, about 250 bytes, and TCP/IP instead is commonly used for constant and large data traffic. Then, the information to be transmitted has to be divided to the NFC payload in as many packets as needed, and all those packets have to include the TCP/IP headers.

An approach was made a while ago to prove that TCP/IP could travel over NFC [25]. In that work, the TCP/IP stack and a substantial part of the communications and NFC procedures were done in an external computer. Then the computer sent the TCP/IP package through Bluetooth to a device where that package was retransmitted through NFC. This work was only a proof of concept that showed NFC could transport IP packets, but the only thing achieved was the inclusion of IP packets in NFC payload, no proper performance of TCP/IP communication over NFC was made. What we want, is to develop a sensor tag with full TCP/IP suite, web server and NFC communication to provide new contactless near field services using standard protocols and web browsers.

Regarding the reader device (mobile phone, laptop, *etc.* equipped with NFC), a software driver will be the responsible of creating a virtual network interface, handle the NFC packets and redirect all the TCP/IP data to that interface, so the TCP/IP traffic is tunneled over the NFC carrier in a transparent way to the user.

### 3.3. Processing Unit

The Central Processing Unit (CPU) is the *heart* of the microcontroller, and the computation capability of the WebTag relies on it. In this case the most restrictive requirement is that it has to be able to host an embedded web server, a downsized server implementation compliant with HTTP protocol [26]. Aside being a well studied cross platform protocol, clients of those web servers are usually web browsers, which are already available in any electronic device with Internet access.

These embedded web servers have some functional limitations depending on the microcontroller memory constraints and the network protocols. As a general rule, memory and CPU utilization should be minimized and the web server should be able to serve different pages from application memory. There it is one of the big challenges of WebTag.

### 3.4. Security

WebTag allows a reduction of the risk related to the malicious wireless access by means of two important design rules, inherited from the near field point-to-point access and IP. The former is simply to reduce the read coverage up to a few centimeters, making the interaction by radio coupling. The latter is a consequence of the use of an embedded web server, as it allows deploying web authentication based methods. Sensor tags usually provide encryption techniques as security method for transmitting data over the air, but if exchanged data contains private or significant information, deploying security systems beyond encryption as authentication methods may be necessary. Implementing a login utility to access WebTag prevents third party users without permission to access the system. In addition, we can keep a record of successful and unsuccessful accesses over time.

### 3.5. Wireless Transmission

Sensor tags usually have small bandwidths, intended to exchange small amount of data (personal identification or sensor data) so they are not prepared for transmitting large information.

For WebTag, HyperText Markup Language (HTML) pages and all the extra data introduced by the used protocols and physical layers would be too large for the radiofrequency channel capabilities. This always means the need to split the data in several parts, increasing the channel usage, reducing the data transmission efficiency (more overheads), and therefore increasing again the overall transmitted data.

This decrease in transmission efficiency affects directly to the user experience if the read range is very short, because the user should be close to the tag for too long. WebTag proposes the use of compression techniques based on the idea that TCP/IP headers do not change very much during a connection and that the format of the data is previously known (HTML syntax).

### 3.6. Power Consumption

Power consumption is among the most significant performance metrics for sensor tags due to the fact they are usually powered by small batteries. Power consumption depends not only on the hardware used, but also on how the hardware is used and how often high computational and memory processes are made by the CPU.

As sensor tags have to remain operational through a determinate amount of time, it is important to use dynamic voltage and frequency scaling, reducing the supply voltage and clock frequency, and dynamic power management techniques, as powering down the unused hardware or peripherals. Moreover, the system should stay in low power modes when possible to save batteries.

## 4. Implementation and Evaluation

**Figure 4.**
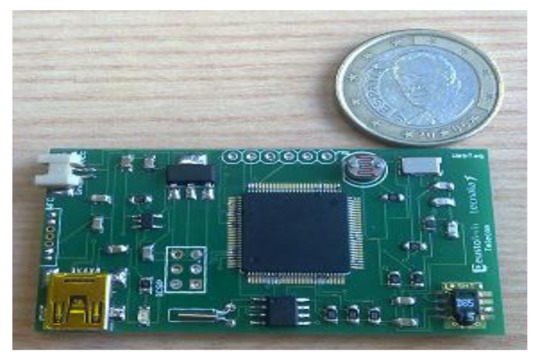
WebTag.

The current WebTag prototype (Figure 4) is based on the ATmega2560 chip (256 KB of flash memory and 8 KB of RAM) with the Arduino bootloader built in it. The prototype has 2 sides: upper side has the chip, the battery connector, the USB-battery charger circuit, the set of sensors and the hardware RTC chip. The bottom side has the NFC chip (NXP PN532), the matching circuit and the antenna. The microcontroller is connected through a pin head to the NFC reader.

### 4.1. Architecture

In this implementation two sensors have been used: digital temperature and humidity sensor (SHT15) and a light dependent resistor (LDR). The LDR is connected to the microcontroller through an analog interface, and the data conditioning is done with internal analog to digital converter (ADC). The SHT15 digital temperature/humidity sensor uses a SPI interface to communicate with the CPU. All the sampled values are stored in memory with the sampling time and date. A hardware Real Time Clock (DS1337) has been used to keep track of the current time, so that the microcontroller has a solid clock reference. For sampling the sensors, an alarm is set in the RTC so the microcontroller wakes up from sleep.

### 4.2. Communication Protocol

WebTag is based on the uIP TCP/IP communication stack. It is an implementation of the TCP/IP protocol stack intended for small 8-bit and 16-bit microcontrollers. It provides the necessary protocols for Internet communication with a very small code footprint and RAM (Random Access Memory) requirements. It is open source and it has been ported to a wide range of 8-bit microcontrollers and it is used in a large number of embedded products and projects.

**Figure 5.**
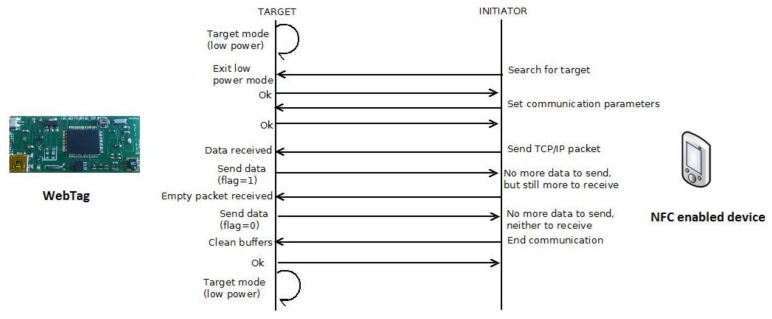
Reduced NFC communication procedure.

At this point, it is important to note how NFC works. In peer-to-peer applications NFC communication procedure follows a master-slave mode, in which one end of the communication acts as the initiator and the other end, in this case WebTag, acts as a target (see Figure 5). Therefore, WebTag enters in the target mode state (low power) and waits till an initiator starts a communication.

Unfortunately, the target can only respond to an initiator if this last one has sent a packet or a request. As TCP/IP traffic does not work like this, as sometimes one end has more data to send than the other one, a specific communication procedure has to be used. In this case, the initiator knows if the target has still information to send by a byte used as a flag in the NFC header. When this flag is on, the initiator sends another packet to the target so all the remaining data can be sent.

Once the communication is ended, the target gets back to its low power state waiting for another initiator request.

### 4.3. Embedded Web Server

WebTag uses a downsized embedded web server that listens on port 80 for incoming HTTP requests and it can handle POST and GET methods. The way it manages the information that has to exchange with the client is based on states. Thus, the embedded web server has a variable which contains the actual state of the server and depending on that state and the request message received from the client side, serves different contents.

Contents are HTML web pages stored in memory. Hence, web server can display different pages, as: login, real time sensors diagram, values of the sensor samplings saved to date with the chance of sending them through POST/GET to an outside server for further processing, an email form and a webpage which displays information about WebTag and also allows modifying certain run time variables, as sampling time.

In order to establish an IP connection with WebTag, a bash script has been developed in Ubuntu environment. This script creates a virtual network interface (matching the IP address of WebTag), reads and processes the packets from the NFC reader (connected via USB) and sends the encapsulated TCP/IP packets to the virtual network interface so the user can surf WebTag in a transparent way.

When a connection is established with WebTag, the embedded web server analyzes every incoming HTTP packet and its actual state to know what content the user is demanding.

To test the proper performance of the web server, the information stored in WebTag can be sent to a remote server. All the stored values are processed in a configurable format to build as many strings as necessary to send the information within the HTML pages. All those values will be visible to the client through its web browser in the specified format, and if desired, the browser can send that information to a remote server through a POST method and its Internet connection for further processing. When the data arrives to the server and it is stored, an acknowledge packet is sent back to the client in order to redirect it to WebTag, and once that confirmation from the remote server has been received, those stored values are erased from memory. The fact of seeing real time sensor values and the chance of sending all the stored values to a remote server through Internet for further processing, all by web browser interaction allows different kind of scenarios and usability.

### 4.4. Security

Keeping unauthorized users from accessing the information is something mandatory in many identification and monitoring systems related with security applications. To avoid any external user to access the information within the web server two methods of security are implemented.

The first one is a ciphering implementation based on ARC4 or ARCFOUR. This encryption system is one of the most used software stream cipher and it is used in popular protocols such as Secure Sockets Layer (SSL) and Wired Equivalent Privacy (WEP). It is very simple and lightweight but has some weaknesses, as the insufficient key schedule, in which the first encrypted bytes reveal some information about the key. If these bytes are discarded, efficiency can be improved. For WebTag we believe this kind of encryption gives us a good compromise between security and computational needs, so we have developed a small encryption implementation based on ARC4. We generate a key stream using a key-scheduling algorithm (KSA) for the permutation of bytes and a pseudo-random generation algorithm (PRGA) to generate the stream.

**Figure 6.**
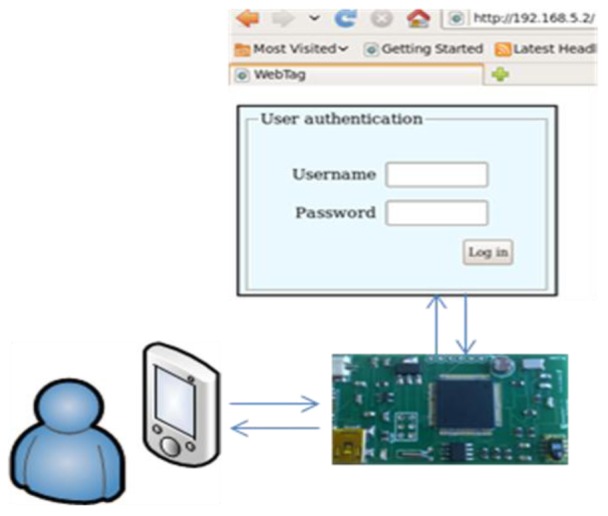
Authentication process.

The WebTag prototype also implements a login access control service in order to avoid any external user to access the information within the web server (Figure 6). If the username and password are not properly introduced in a configurable number of attempts, the system will not allow connections for a time and will set a warning flag in memory with the time and date of the failed access attempt to be reported when the tag is surfed. This kind of security is not implemented in the rest of point-to-point access sensor tags and allows having a log of accesses ordered by date.

### 4.5. Wireless Transmission

The NFC module is connected to one UART in the CPU. Every time a packet arrives, the microcontroller gets the TCP/IP header and payload and sends it to the IP stack for processing. After a response, the packet is compressed if possible (header and data), the NFC header is calculated and sent to the NFC module for wireless transmission.

Although we have tried different maximum transmission units (MTU), we have noticed that sometimes the response time is not as quick as desired, which is understandable.

The used NFC chip has a receive and transmit buffer of only 64 bytes that can be processed and transmitted at once, so a bottleneck problem could happen if we try to send more data at once. Besides, the master-slave working mode of NFC forces us to send some control packets in order to ensure the whole information exchange, increasing the channel usage and its latency.

On the other hand, the path those packets have to travel between the NFC device, the CPU and the internal processes, their different processing speeds, among the several partly buffers for data processing increased the transmission latency.

That is why reducing the size of the headers of those packets flowing through the wireless link provides many positive aspects to data exchange, like an improvement of the response time seen by the user. The compression allows the use of small packets (suitable for interactive traffic) without affecting the efficiency of the bandwidth of the channel.

Header compression is especially desirable in environments where links have low values of the MTU, as NFC. This feature responds to the need to minimize the error probability of the packets flowing through the network.

Considered the power and memory limitations, it is mandatory to implement a low profile header compression. We have developed a compression algorithm based on Van Jacobson compression due to its greater simplicity compared to other standards. This standard is based on delta coding, which transmits only the differences between successive packets. The algorithm is based on two characteristics of TCP/IP connection traffic:

- Some of the fields of the header remain invariant during a connection.
- Another fields change a predictable value.

Then, the fields which vary along the connection are replaced by increments of the same field of the immediately preceding segment. For example, the sequence number of each received segment is calculated from the previous segment by adding the incremental value indicated by the new segment. Keeping header buffers for both incoming and outgoing packets allows us to increase the compression range by calculating some fields from both headers. After a compressed packet is sent, the header buffer is updated with the new values.

Following this implementation we have been able to achieve a compression rate of 87.5%, sending only 5 bytes of TCP/IP header, sufficient to convey all the necessary information, like the total length, TCP checksum and flag or the ACK number increment. After a connection is set free, header buffers are cleaned and next time a packet arrives (SYN) the process starts all over again.

### 4.6. Power Consumption

The power consumption of WebTag is shown in Table 2. These values have been obtained with a 3.7 V external LiPo battery.

Power management efforts have been focused in minimizing CPU, memory and peripheral device usage by implementing some energy management software techniques and using low power modes. In order to avoid wasting power, the peripherals that are not used, like different timers and interfaces, are powered down permanently, but the ones that are used only in short periods are powered down till they are requested for activation. Thus, a considerable amount of power is saved in large periods. When there is no web server activity the NFC device and the CPU enter low power modes. Only two external interrupts can wake up again the system: NFC initiator request and the hardware RTC triggering.

**Figure 7.**
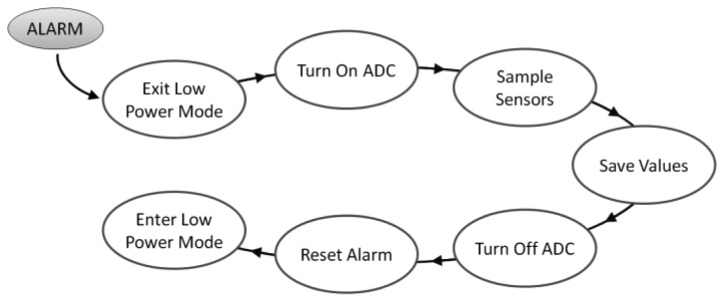
Sensor sampling procedure.

A configurable alarm based on the RTC is the responsible of triggering the sensor samplings. When it is time to check the sensors, the CPU exits from the low power mode and turns on the ADC module to perform the conversion. After sensors are sampled, the current values are compared with the previous values and if they do not differ a configurable percentage, they are not saved in memory, reducing CPU and memory usage. Then, the ADC is powered down and the alarm is set again into the RTC to wake the CPU when it is time to sample the sensors again (see Figure 7). After setting the alarm, the system enters again into the sleep mode, which will be more or less deep regarding on how long the alarm is set, so a compromise between low power mode and proper sampling time is desirable.

## 5. Conclusions

This paper introduces WebTag, a new concept of smart sensor tags with web access. It is based on the near field (NFC) point-to-point technology over a micro embedded web server. It represents a step forward to the evolution of the Internet—from a simple inter-computer connection to an Internet of Things paradigm—applied to the wireless sensor monitoring for secure applications.

WebTag tries to merge the advantages of the NFC and IP technologies, *i.e.*, point-to-point and web access, creating a new access method for wireless sensor monitoring. A TCP/IP enabled sensor allows a bidirectional data transfer whereby read and write operations are possible with standard web browsers, extending the number of readers to any device with that application inside. Moreover, all the capabilities available in the Internet are used by WebTag in order to increase the security of data transmission and to provide a user login.

Unless WebTag has been validated in a near field point-to-point application, this is the first step to its integration in wireless sensor networks. This work shows the challenges of integrating a web access into a sensor tag. On the one hand, we found some of the problems related with the low power requirements. All the hardware and software designs need to be optimized in power consumption, and the inclusion of web based communication technologies in low power microcontrollers has been solved successfully.

On the other hand, the fact of providing a web access over radiofrequency identification technology has been a major challenge. This is because NFC and TCP/IP differ in their basic way of functioning. TCP/IP is prepared for the exchange of large and constant amount of data, while NFC is prepared for small payloads, few exchanges and a master-slave model. Thus, enabling an acceptable TCP/IP tunnel over NFC in terms of latency, number of exchanges and memory usage has been fulfilled also successfully.

The paper remarks the critical design guidelines and implementation details, and the final validation as a functional web based access over NFC: the stored data is sent to a remote server for data processing through the Internet access of the NFC reader in a transparent way for the end user. We expect WebTag to be the first step of a future sensor tag generation with new interaction capabilities and more tracking and monitoring application opportunities.

## Figures and Tables

**Table 1.** RFID *vs.* NFC *vs.* Bluetooth.

	**RFID**	**NFC**	**Bluetooth**
***Network type***	Point-to-point	Point-to-point	WPAN
***Communication***	Unidirectional	Bidirectional	Bidirectional
***Security***	Hardware and protocol level	Hardware and protocol level	Protocol level
***Range***	Up to 100 m	<0.2 m	∼100 m (class 1)
***Frequency***	LF/HF/UHF/Microwave	13.56 MHz	2.4–2.5 GHz
***Bit rate***	Varies with frequency	Up to 424 kbit/s	2.1 Mbit/s
***Set-up time***	<0.1 s	<0.1 s	<6 s
***Power consumption***	Varies with frequency	<15 mA	Varies with class
***Continuous sampling***	No	Yes	Yes

**Table 2.** Power consumption.

**Mode**	**mA**
Active	15–20
Low power	1
Active and NFC usage	40
